# SGnn: A Web Server for the Prediction of Prion-Like Domains Recruitment to Stress Granules Upon Heat Stress

**DOI:** 10.3389/fmolb.2021.718301

**Published:** 2021-08-18

**Authors:** Valentín Iglesias, Jaime Santos, Juan Santos-Suárez, Carlos Pintado-Grima, Salvador Ventura

**Affiliations:** Institut de Biotecnologia i Biomedicina and Departament de Bioquímica i Biologia Molecular, Universitat Autònoma de Barcelona, Bellaterra, Barcelona, Spain

**Keywords:** stress granules, prion-like domains, protein aggregation, yeast prions, bioinformatics, machine learning

## Abstract

Proteins bearing prion-like domains (PrLDs) are essential players in stress granules (SG) assembly. Analysis of data on heat stress-induced recruitment of yeast PrLDs to SG suggests that this propensity might be connected with three defined protein biophysical features: aggregation propensity, net charge, and the presence of free cysteines. These three properties can be read directly in the PrLDs sequences, and their combination allows to predict protein recruitment to SG under heat stress. On this basis, we implemented SGnn, an online predictor of SG recruitment that exploits a feed-forward neural network for high accuracy classification of the assembly behavior of PrLDs. The simplicity and precision of our strategy should allow its implementation to identify heat stress-induced SG-forming proteins in complete proteomes.

## Introduction

Biomolecular condensates are a group of diverse membraneless organelles formed by the association of proteins that undergo liquid to liquid or liquid to solid phase transitions in the cellular milieu (Banani et al., 2017; Woodruff et al., 2018; Shiina, 2019). Stress granules (SG) are a subclass of biological condensates which form in response to different cellular stresses and disassemble when the stress is released, in a dynamic and highly regulated process involving liquid-liquid phase separation (LLPS) reactions (Protter and Parker, 2016; Mahboubi and Stochaj, 2017). They are constituted by selected proteins and mRNAs stalled in translation initiation (Protter and Parker, 2016). A significant fraction of these proteins contains prion-like domains (PrLDs), which are key regulators of phase transitions (Boeynaems et al., 2018). PrLDs are low complexity and intrinsically disordered protein regions with a compositional bias resembling that of the prion domains (PrDs) of yeast prions, which also experiment phase transitions from initially soluble to aggregated states (King et al., 2012; Wickner et al., 2015).

In a recent work, Ross and coworkers studied the recruitment of a set of *Saccharomyces cerevisiae* PrLDs into SG when the cells were heat-stressed (Boncella et al., 2020). They demonstrated that the PrLDs recruited to these granules differed in composition from those that did not show stress-dependent assembly. Positive PrLDs showed enrichment in hydrophobic residues (WFILV), charged residues (HEKR), and cysteine. These compositional biases were surprising since they do not fit with the properties that make these sequences prion-like (Alberti et al., 2009; Toombs et al., 2012; Sabate et al., 2015). Classical yeast PrDs are generally enriched in Q/N residues and depleted in hydrophobic and charged residues. These compositional differences were used to create an algorithm to identify PrLDs that would assemble into stress foci in response to heat shock. This tool allowed them to manipulate the recruitment of preexisting PrLDs and the *de novo* design of synthetic PrLDs with a selected response to heat stress (Boncella et al., 2020).

We noticed that Phe and Trp, but not Tyr, were overrepresented in positive PrLDs. This observation was unexpected because Tyr is the only enriched aromatic amino acid in the sequence of PrLDs (Sabate et al., 2015), and it had been proven to be a fundamental driver of LLPS by establishing multivalent cation-π interactions with arginine residues (Wang et al., 2018; Murthy et al., 2019), a role that Phe cannot replace (Wang et al., 2018; Batlle et al., 2020). Besides, synthetic PrLDs revealed that aromatic residues were dispensable for the assembly of SG, since aliphatic ones could replace them. The secondary role played by aromatic residues in these reactions, especially in the case of Tyr, suggests that a mechanism alternative to LLPS might be behind the observed PrLDs intracellular assembly upon heat stress. An extensive proteomic analysis performed by Wallace and coworkers in *S. cerevisiae* suggested that a reversible aggregation mechanism controls the formation of SG, acting as a cellular adaptation to thermal stress (Wallace et al., 2015). From our perspective, it was tempting to speculate that such an aggregation-based mechanism might be responsible, at least in part, for the stress-induced assembly reported by Ross and coworkers, justifying the insensitivity of the process to aromatic to aliphatic mutations and the bias towards classical aliphatic aggregation-prone residues, which are otherwise strongly underrepresented in PrLDs (Alberti et al., 2009; Sabate et al., 2015). Under this premise, we tried to dissect the particular biophysical features behind the heat stress-induced assembly of PrLDs into SG, and we developed SGnn, a neural network-based prediction method able to discriminate PrLDs assembly by evaluating their aggregation propensity, net charge, and disulfide bonding potential. The SGnn web server is freely available for academic users at http://sgnn.ppmclab.com.

## Methods

### Evaluation of the molecular determinants responsible for PrLDs recruitment to SG

The set of 69 natural and synthetic prion-like domains described by Ross and coworkers in *Saccharomyces cerevisiae* was analyzed using AGGRESCAN (Conchillo-Sole et al., 2007) and CamSol Intrinsic (Sormanni et al., 2015) algorithms to evaluate protein aggregation propensities. The Henderson-Hasselbalch equation was employed to calculate the net charge per residue (NCPR). Cysteine content was computed by evaluating the frequency of this residue in the sequences.

### Dataset Description

Following the original article, natural, synthetic, and redesigned PrLDs were clustered according to their tendency to assemble into stress foci after 30 min of heat shock at 46°C in three datasets: 1) PrLDs that formed foci in ≥60% of cells (*n* = 32) were considered positive, 2) negative were those PrLDs assembled in less than a 25% of the cells (*n* = 32) and 3) PrLDs that range from a 26 to a 59% (*n* = 5) were classified as intermediate. A two-tailed Mann-Whitney test was used to compare the average scores for positive and negative datasets (Table 1).

**TABLE 1:** Mean values for aggregation, NCPR, and cysteine percentage box-plots in positive, intermediate, and negative datasets represented in Figure 1 *p*-values for the differences between the positive and negative PrLDs subsets are shown.

Dataset	Aggregation		NCPR (x100)	Cysteine (%)
	AGGRESCAN	CamSol Intrinsic		
**Positives**	-17.175	1.245	3.09	1.5
**Intermediate**	-30.84	1.645	1.35	0.28
**Negative**	-42.112	2.017	-0.795	0.184
*p* **-value**	**<0.0001** ^a^	**<0.0001** ^a^	**0.2921**	**0.0002** ^a^

a *p*-values < 0.001 were considered to be statistically significant.

### Performance Analysis

The precision of the different predictive methods was evaluated using a ROC analysis, in which the true-positive rate is plotted against the false-positive rate for the *in vivo* obtained positive and negative datasets.

Binary classification performance was evaluated attending to their sensitivity, specificity, precision, accuracy, F1 Score and Matthews Correlation Coefficient (MCC) as follows: Sensitivity = TP/(TP + FN); Specificity = TN/(TN + FP); Precision = TP/(TP + FP); Accuracy = (TP + TN)/(TP + TN + FP + FN); F1 Score = TP/(TP + ½(FP + FN)) and MCC = (TP*TN - FP*FN)/[(TP + FP) (TP + FN) (TN + FP) (TN + FN)]^1/2^. TP, TN, FP and FN correspond to true positives, true negatives, false positives and false negatives, respectively.

### Training of the Feed-Forward Neural Network (FFNN) for the Binary Classification of PrLDs

To develop a predictive strategy based on the distinct properties observed in *vivo* PrLDs recruited to SG, we trained an FFNN to anticipate PrLDs behavior based on their aggregation propensity, NCPR, and cysteine percentage. For the training, we randomly segregated 50% of the PrLDs from the positive and negative datasets (16 positives and 16 negatives PrLDs). AGGRESCAN aggregation propensity, NCPR, and cysteine percentage were calculated as described above. The FFNN was created using version 0.3.5 of the neurolab Python package and consists of a multilayer-perceptron network with three inputs, nine neurons in the input layer, six neurons in the hidden layer, and one in the output layer. Optimization was performed using the gdx algorithm (gradient descent with momentum and adaptative learning rate backpropagation), which combines adaptative learning rate with momentum training. Once trained, FFNN performance was tested against the remaining 16 positive, and 16 negative sequences and its discriminatory potential was evaluated. To exclude potential biases associated with the random configuration of the datasets, we repeated the training and testing with diverse randomizations that resulted in very similar overall classifications.

### SGnn Implementation

For each input PrLD sequence, SGnn calculates AGGRESCAN aggregation propensity, NCPR, and Cysteine percentage. AGGRESCAN “Na4vSS” scores include positive and negative values; therefore, these were normalized between the most and least aggregation-prone natural PrLD (Supplementary Table S1) to feed the neural network only with positive aggregation propensity-values. Finally, the obtained numerical results are fed to the FFNN, which establishes a binary outcome.

### SGnn Web Server

SGnn web server is platform-independent and has been tested in modern browsers. Its interface was built in a combination of HTML, CSS, and JavaScript. SGnn uses the Django 3.0 framework working with Python 3.7.5. SGnn back-end script was written in Python. The web server includes detailed documentation and a pre-loaded example of positive and negative PrLDs. The output figure is generated using the matplotlib library (Hunter, 2007).

## Results

### Computational Analysis of the Molecular Determinants Driving Prion-Like Domains Assembly

We explored different physicochemical features that might potentially contribute to the intracellular heat-induced assembly of yeast PrLDs.

**FIGURE 1:**
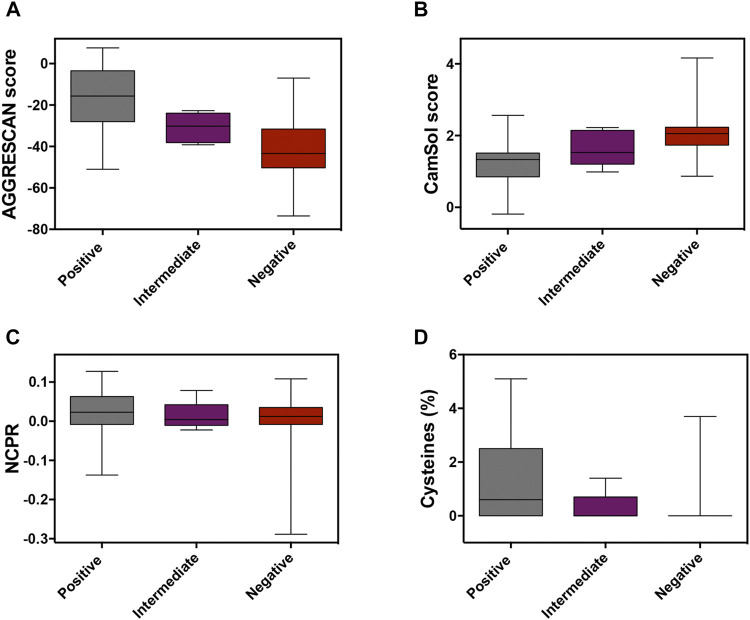
Analysis of the molecular determinants involved in prion-like domains (PrLDs) recruitment to stress granules (SG). Box-plots depicting different properties of assembling and non-assembling PrLDs in the complete dataset: protein aggregation using **(A)** AGGRESCAN and **(B)** CamSol Intrinsic predictors, **(C)** the net charge per residue, and **(D)** the cysteine percentage.

In globular proteins, hydrophobic residues are usually protected from the solvent in the inner core, and their exposure by unfolding is connected to protein aggregation (Kelly, 1998). PrLDs have a disordered nature, and therefore hydrophobic residues in their sequences are necessarily exposed and ready to establish contacts with other lipophilic amino acids, which may ultimately result in protein aggregation. The unexpected enrichment in hydrophobic residues in positive PrLDs in the above-described dataset immediately suggested that, in line with previous observations (Wallace et al., 2015), protein aggregation might be, somehow, behind heat-induced stress granule formation. We addressed the role of the sequence aggregation propensity using two algorithms based on different prediction principles: AGGRESCAN and Camsol intrinsic (Figures 1A,B; Supplementary Table S1). Both programs predicted the positive PrLDs in the dataset to be significantly more aggregation-prone than negative PrLDs (Table 1), thus suggesting a relationship between the assembly of PrLDs into SGs and their aggregation tendency.

Ross and coworkers identified enrichment in ionizable residues in positive PrLDs, suggesting that charges may play a role in the assembly of SG. The charge of PrLDs might influence this reaction in two different ways: 1) First, heterotypic electrostatic protein-protein interactions have been described as drivers of LLPS (Mitrea and Kriwacki, 2016) and 2) positively charged residues are fundamental for the interaction with the negatively charged RNA recruited into these stress foci (Law et al., 2006). We analyzed if the net charge per residue (NCPR) of PrLDs might somehow influence its ability to form SG (Figure 1C; Supplementary Table S1). On average, recruited PrLDs tend to be positively charged, whereas PrLDs from the negative dataset are slightly anionic (Table 1). Anionic and cationic residues contribute equally to intermolecular electrostatic interactions. Thus, the higher prevalence of cationic residues in positive PrLDs likely results from their specific ability to interact with RNA molecules. The differences in NCPR between positive and negative PrLDs were evident but not statistically significant, likely because whereas negatively charged residues cannot contribute to nucleic acid binding, they are necessary for ionic protein-protein interactions.

Cysteine was found to be enriched in the set of positive PrLDs (Boncella et al., 2020). Again, this observation was surprising because this residue is known to be strongly underrepresented in PrLDs sequences (Alberti et al., 2009; Toombs et al., 2012; Sabate et al., 2015). Since heat stress has been directly associated with the accumulation of reactive oxygen species (Flanagan et al., 1998), it seems plausible to speculate that the oxidation of Cys thiol groups in PrLDs can be relevant for the assembly of SG. This reaction has already been described as a trigger of TDP-43 recruitment to SG (Liu-Yesucevitz et al., 2010; Cohen et al., 2012; Dewey et al., 2012). Either the formation of covalent links (Cumming et al., 2004), that would stabilize protein-protein interactions in SG, or the oxidation of cysteine to sulfenic or sulfonic acids (Hamann et al., 2002), modifications reported to accelerate protein aggregation (Marinelli et al., 2018), may be possible explanations for Cys overrepresentation in PrLDs recruited to SG. Our analysis (Figure 1D) indicates that the enrichment in Cys of positive PrLDs, relative to their negative counterparts, is, indeed, statistically significant (Table 1).

**FIGURE 2:**
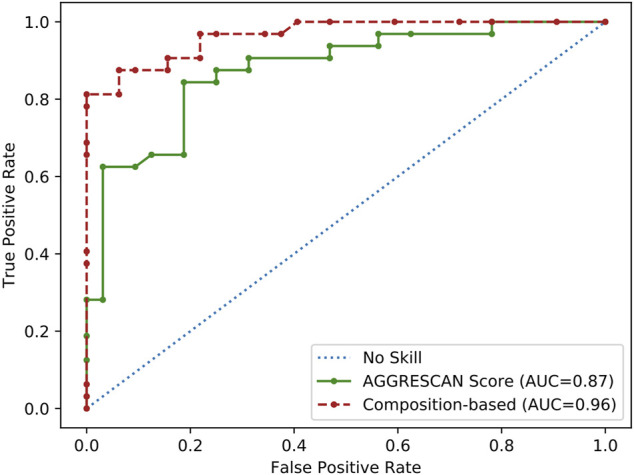
ROC curve analysis of the aggregation propensity as a predictor of PrLDs assembly propensity. Performance of aggregation propensity and composition predictions of heat-induced PrLDs *in vivo* recruitment to SG as described by Ross and coworkers (Boncella et al., 2020).

Overall, our analysis suggested that the observed compositional bias in SG-forming PrLDs might stem from a combination of at least three physicochemical: an increased sequential aggregation propensity, the ability to establish electrostatic interactions, and the possibility to form disulfide bonds. Aggregation propensity seems to be a particularly important determinant of heat-induced foci formation since AGGRESCAN alone discriminated reasonably well positive and negatives PrLDs when analyzing the complete dataset (*n* = 64), according to the derived ROC curve (AUC = 0.87), approaching the performance of the tailor-made composition-based approach (AUC = 0.96) (Figure 2).

### Development and Implementation of SGnn, a Machine Learning Strategy for Predicting PrLDs Heat-Induced Recruitment to Stress Granules

Based on the evidence that positive PrLD seemed to possess distinct physicochemical features, we aimed to build up a novel SG predictor.

To that end, we needed to define a relationship between the three variables that would allow an accurate binary classification between two states, corresponding to assembly-competent and assembly-incompetent sequences. We could not assume linear correlations exist nor develop tentative modeling equations from scratch, which precluded the use of classical iterative analysis relying on variables parametrization.

To bypass these limitations and exclude arbitrary assumptions, we decided to use a supervised machine learning approximation able to recreate non-linear models based on a multi-layer perceptron FFNN. We randomly segregated the experimentally characterized PrLDs (32 positive and 32 negative instances) in two datasets with an equal number of positive and negative sequences. One dataset was used to train a Feed-Forward Neural Network (FFNN) to project those three input features into a binary classification of positive and negative PrLDs. The second dataset was used to test the performance of the FFNN. In the test dataset, 14 of the analyzed sequences were classified as true positives, 16 as true negatives, and 2 as false negatives, which resulted in an excellent performance as evaluated by the sensitivity, specificity, F1 score, and Mathews correlation coefficient (Table 2). Very similar results were obtained using other random configurations of the training and testing sets (not shown). When we analyzed the complete dataset of PrLDs, we obtained similar performances (Table 2; Supplementary Table S1), outperforming those of the tailor-made composition-based approach.

**TABLE 2:** Performances of the composition-based and SGnn approaches in predicting heat-induced PrLDs recruitment to SG.

	Composition-based	SGnn	
		Testing set	Complete dataset
**Specificity**	0.97	1	0.97
**Sensitivity**	0.78	0.88	0.88
**Accuracy**	0.88	0.94	0.92
**Precision**	0.82	0.88	0.89
**F1 Score**	0.89	0.94	0.93
**Matthews correlation coefficient**	0.76	0.88	0.84

**FIGURE 3:**
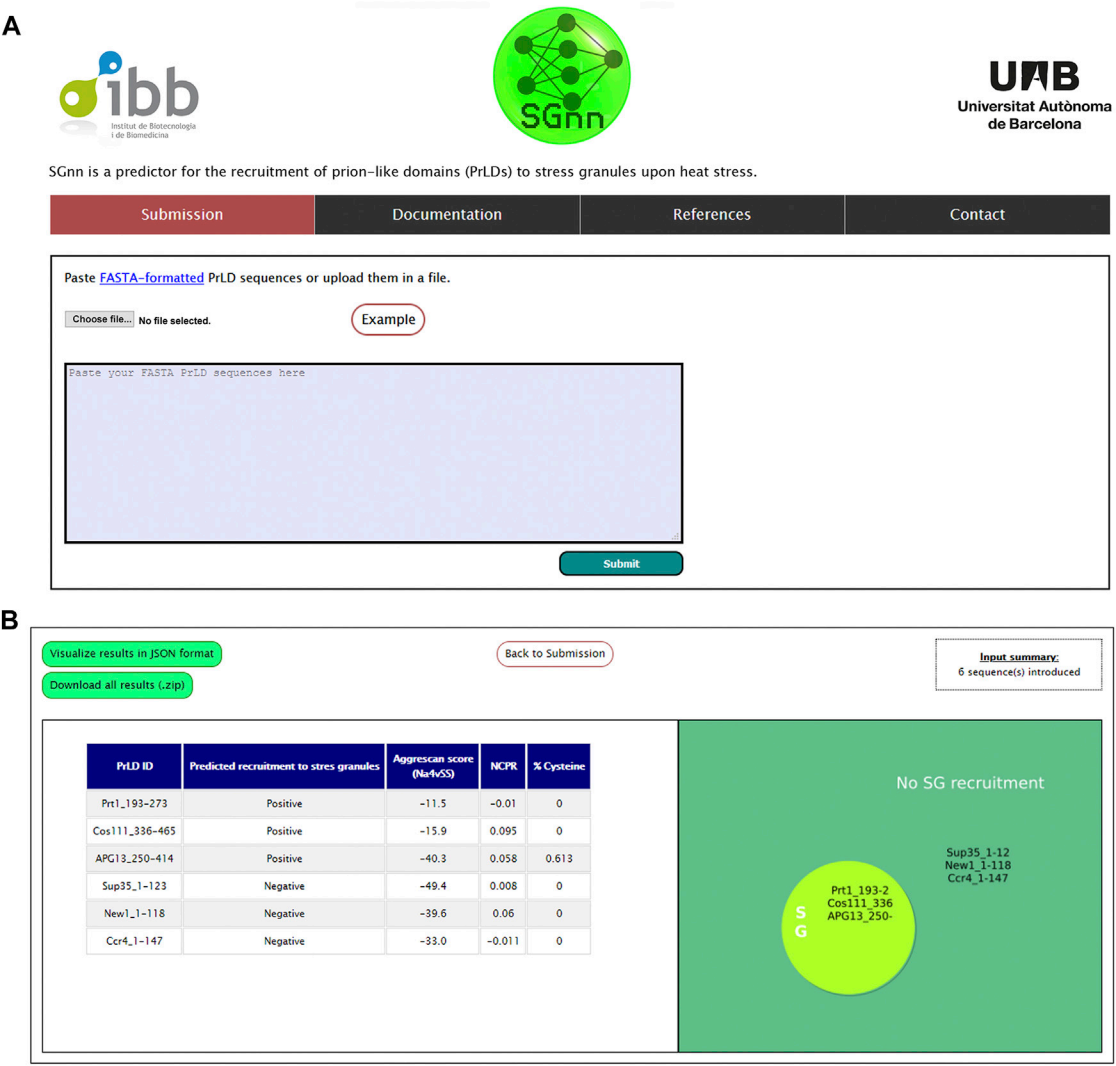
SGnn web server interface. **(A)** Web input page. Users can input their sequences in the provided window or upload them in FASTA format. **(B)** Overview of SGnn results page. On completion, SGnn will show the results in a table format and a figure summarizing the prediction. Alternatively, users can click the upper links to retrieve them in JSON or ZIP format.

Based on our FFNN, we next aimed to build an online computational tool to predict PrLDs recruitment to SG upon heat stress, which we named SGnn. The algorithm computes the AGGRESCAN aggregation propensity, NCPR, and cysteine percentage of one or multiple input sequences and exploits our FFNN to classify those PrLD sequences as positive or negative. SGnn, which is available at http://sgnn.ppmclab.com is free for academic users and does not require previous login. On the input page, users can introduce one or multiple PrLD sequences in FASTA format or upload them in a single file (Figure 3A). Alternatively, users can pre-load example sequences to test SGnn. After running SGnn, the AGGRESCAN score, NCPR, cysteine percentage and FFNN prediction will be available on the results page, as shown in Figure 3B. Users can retrieve the results in a JavaScript Object Notation (JSON) formatted file or download them as a compressed ZIP folder where all the project-generated data is available.

Of note, SGnn was trained on top of pre-defined yeast PrLDs and, accordingly, it is not intended to be used in full-length proteins or regions that do not possess prion-like features. We encourage users to pre-scan their dataset with dedicated software that searches for sequence similarity to yeast prions such as PLAAC, PrionScan, PAPA or PrionW in order to delimit the proteins’ PrLDs (Toombs et al., 2012; Espinosa Angarica et al., 2014; Lancaster et al., 2014; Zambrano et al., 2015). We also recommend users to read the documentation page, where practical instructions for the use of SGnn are provided.

## Discussion

Protein composition-based strategies have shown to be accurate in predicting the assembly behavior of PrLDs in front of heat stress (Boncella et al., 2020). However, composition alone is a black box from which it is difficult to decipher the mechanistic rules behind the observed phenomenon. The advantage of decoding these properties is double as it: 1) allows for a rationalization of the observations, i.e., PrLDs with very soluble sequences would rarely form heat stress-induced SG, and 2) facilitates redesign and *de novo* design ventures, as illustrated by the higher performance of our approach in forecasting the properties of synthetic PrLDs, even if they were generated to fit the composition-based model of SG formation (Supplementary Table S2).

Cation-π interactions between Tyr and Arg are considered important contributors to the multivalent interactions driving LLPS processes. In fact, in FUS family proteins, the number of Tyr and Arg within the PrLDs is sufficient to anticipate their LLPS propensity (Wang et al., 2018). In contrast, in the dataset of PrLDs recruited to SG generated by Ross and coworkers, Tyr is underrepresented, and aromatic to aliphatic substitutions do not interfere with SG recruitment. In our opinion, this suggests that the observed PrLDs intracellular assembly may be governed by physicochemical features distinct from those conventionally associated with the LLPS of FUS-related proteins. Our analysis suggest that an aggregation-related mechanism might be a more appropriate descriptor of PrLDs coacervation upon heat stress in yeast.

Our results indicated that three simple biophysical properties, namely the aggregation propensity, the net charge, and the cysteine content, might suffice to describe the heat-induced assembly of PrLDs into SG. Driven by this evidence and using *in vivo* derived data, we developed SGnn, a machine learning strategy dedicated to evaluate the heat-induced assembly of PrLDs in SG, which is freely available for academic users. Protein aggregation depends on the presence of defined aggregation-prone regions reactions that nucleate the self-assembly and, ultimately, in the specific protein sequence. In contrast, the NCPR and the Cys percentage are composition-related terms. Thus, it seems that a combination of sequence- and composition-dependent features provides the best prediction of the propensity of a PrLD to be recruited into SG upon heat stress. An observation similar to the one we reported previously for the formation of pathogenic intracellular foci by PrLD-containing proteins (Batlle et al., 2017; Iglesias et al., 2019).

SGnn is a new computational tool dedicated to the prediction of PrLD recruitment to heat-induced SG, which as most algorithms devoted to studying prion-like properties, has been developed using yeast-derived data (Toombs et al., 2012; Lancaster et al., 2014; Sabate et al., 2015; Zambrano et al., 2015). As for them, we expect SGnn predictions to be transferable to other species, becoming a valuable tool for the identification of SG forming prion-like proteins in large protein datasets, including the characterization of the human heat stress-induced granulome or the identification of proteins that might coalesce into stress granules in fever episodes, both in the human host and in pathogenic viral, bacterial, protozoic or fungal proteomes. Yet, we must note that the use of SGnn requires the previous identification and delimitation of PrLDs in the organism of interest. The compositional traits of these domains might not necessarily coincide in evolutionarily distant organisms, and adapting PrLDs predictions to the proteome of interest is a requirement to obtain context-relevant SGnn forecasts. For these studies, the use of algorithms like PLAAC that consider the proteome compositional background in their PrLDs predictions (Lancaster et al., 2014) is advised. Overall, we envision SGnn as a web server that might help to gather novel insights on the biology and pathology of SG formation in eukaryotic cells.

## Data Availability

The original contributions presented in the study are included in the article/Supplementary Material, further inquiries can be directed to the corresponding author.

## Abbreviations

PrLD, prion-like domain; LLPS, liquid-liquid phase separation; NCPR, net charge per residue; AUC, area under the curve; ROC, receiver operating characteristic curve; SG, stress granules; PrD, prion domains; FFNN, feed-forward neural network.
