# Supplementary material for: SGnn: A Web Server for the Prediction of Prion-Like Domains Recruitment to Stress Granules Upon Heat Stress
Source: Front Mol Biosci. 2021 Aug 18;8:718301. doi: 10.3389/fmolb.2021.718301 (PMC8416484; doi:10.3389/fmolb.2021.718301)
Supplement: Supplementary file 1 [file DataSheet1.docx]

**Supplementary Table S1:** List and score of the PrLDs analyzed in this work. Redesigned and synthetic PrLDs are shown in italics.

| **PrLD** | **Heat-shock response** | **Aggrescan (Na4vSS)** | **NCPR** | **Cysteine (%)** | **Composition-based score** | **Predicted to be recruited by SGnn** |
| --- | --- | --- | --- | --- | --- | --- |
| AI3 (228-387) | Positive | 1.1 | 0.039 | 0.6 | 0.1 | Yes |
| Apg13 (250-414) | Positive | -40.3 | 0.058 | 0.6 | -0.05 | Yes |
| Bem2 (1800-1880) | Intermediate | -30.2 | 0.078 | 0 | 0.22 | - |
| Ccr4 (1-147) | Negative | -33 | -0.011 | 0 | -0.18 | No |
| Cdc39 (966-1092) | Positive | -22.3 | -0.021 | 0 | 0.01 | No |
| Cdc73 (283-393) | Positive | -5.1 | 0.022 | 0.9 | 0.27 | Yes |
| Cln2 (362-503) | Intermediate | -22.7 | 0.001 | 1.4 | 0.04 | - |
| Cos111 (336-465) | Positive | -15.9 | 0.095 | 0 | 0.18 | Yes |
| Crz1 (15-179) | Negative | -43.6 | -0.047 | 0 | -0.04 | No |
| Dat1 (102-236) | Intermediate | -25.1 | 0.004 | 0 | -0.07 | - |
| Ded1 (1-97) | Positive | -44.1 | 0.064 | 0 | 0.02 | No |
| Fab1 (427-552) | Intermediate | -37 | 0.006 | 0 | 0.05 | - |
| Gis1 (454-584) | Negative | -23.7 | 0.009 | 0 | -0.05 | No |
| Grr1 (3-167) | Intermediate | -39.2 | -0.022 | 0 | 0.03 | - |
| Hrk1 (483-647) | Negative | -65 | -0.110 | 3.7 | -0.02 | No |
| Hrr25 (395-494) | Negative | -56.6 | 0.010 | 0 | -0.34 | No |
| Mca1 (1-104) | Negative | -39.9 | 0.010 | 0 | -0.39 | No |
| Mex67 (1-95) | Negative | -7 | 0.023 | 0 | 0.07 | No |
| Mfg1 (1-96) | Negative | -22.4 | -0.010 | 0 | -0.3 | No |
| New1 (1-118) | Negative | -39.6 | 0.060 | 0 | -0.16 | No |
| Pam1 (617-756) | Negative | -31.1 | 0.066 | 0.7 | -0.02 | No |
| Pin4 (169-492) | Negative | -22.3 | 0.009 | 0.3 | -0.02 | No |
| Prt1 (193-273) | Positive | -11.5 | -0.010 | 0 | 0.21 | Yes |
| Pub1 (243-327) | Negative | -46.1 | 0.072 | 0 | -0.27 | No |
| Rpi1 (192-306) | Positive | -31.3 | 0.028 | 0.9 | 0.05 | No |
| RSC8 (232-312) | Positive | -24.8 | 0.017 | 5.1 | 0.22 | Yes |
| Siz1 (390-554) | Positive | -51 | -0.1374 | 2.5 | 0.12 | Yes |
| Sky1 (353-491) | Positive | -33.1 | 0.054 | 2.9 | 0.17 | Yes |
| Sro9 (160-256) | Negative | -67.3 | 0.108 | 0 | -0.14 | No |
| Sup35 (1-123) | Negative | -49.4 | 0.008 | 0 | -0.4 | No |
| Tbs1 (898-1062) | Negative | -73.5 | -0.289 | 0 | 0.03 | No |
| Tda7 (513-636) | Negative | -33 | 0.0008 | 0 | 0.03 | No |
| Tif4631 (1-131) | Negative | -43.3 | 0.033 | 0 | -0.16 | No |
| Trm1 (286-366) | Positive | -2.1 | 0.127 | 2.5 | 0.18 | Yes |
| Ubp3 (1-97) | Negative | -19.3 | -0.008 | 0 | -0.22 | Yes |
| Vac14 (690-818) | Positive | -2.9 | -0.0054 | 1.6 | 0.2 | Yes |
| Vac7 (377-541) | Negative | -40.1 | 0.014 | 0 | -0.01 | No |
| Yck2 (369-533) | Negative | -63.8 | 0.036 | 0 | -0.24 | No |
| *sPrLD2* | Positive | -19.2 | 0.023 | 0 | 0.16 | Yes |
| *sPrLD3* | Positive | -17 | 0.023 | 0 | 0.16 | Yes |
| *cPrLD1* | Negative | -45.5 | -0.008 | 0 | -0.2 | No |
| *cPrLD2* | Negative | -45.5 | -0.008 | 0 | -0.2 | No |
| *sPrLD2 ∆FWY* | Negative | -39.4 | 0.026 | 0 | 0.13 | No |
| *sPrLD3 ∆FWY* | Negative | -36.8 | 0.026 | 0 | 0.13 | No |
| *sPrLD2 FWY→QN* | Negative | -48.6 | 0.023 | 0 | 0.059 | No |
| *sPrLD3 FWY→QN* | Negative | -47.6 | 0.023 | 0 | 0.059 | No |
| *sPrLD2 FWY→ILV (0.18)* | Positive | -17.8 | 0.023 | 0 | 0.18 | Yes |
| *sPrLD3 FWY→ILV* | Positive | -15.4 | 0.023 | 0 | 0.18 | Yes |
| *Prt1 + to –* | Negative | -50.6 | -0.034 | 0 | -0.18 | No |
| *Trm1 + to –* | Negative | -30.4 | 0.039 | 1.2 | -0.16 | No |
| *Rsc8 + to –* | Negative | -54.8 | 0.029 | 0 | -0.16 | No |
| *Mfg1 – to +* | Positive | 7.6 | -0.001 | 0 | 0.15 | Yes |
| *Pub1 – to +* | Positive | -12.8 | 0.059 | 0 | 0.16 | Yes |
| *Sro9 – to +* | Positive | -26.5 | 0.085 | 0 | 0.23 | No |
| *Prt1 Scramble_1* | Positive | -10.2 | -0.022 | 0 | 0.21 | Yes |
| *Prt1 Scramble_2* | Positive | -13 | -0.022 | 0 | 0.21 | Yes |
| *Prt1 Scramble_3* | Positive | -12.9 | -0.022 | 0 | 0.21 | Yes |
| *Prt1 Scramble_4* | Positive | -14.1 | -0.022 | 0 | 0.21 | Yes |
| *Trm1 Scramble_1* | Positive | -0.7 | 0.113 | 2.5 | 0.18 | Yes |
| *Trm1 Scramble_2* | Positive | 0.2 | 0.113 | 2.5 | 0.18 | Yes |
| *Trm1 Scramble_3* | Positive | -0.7 | 0.113 | 2.5 | 0.18 | Yes |
| *Trm1 Scramble_4* | Positive | 0.8 | 0.113 | 2.5 | 0.18 | Yes |
| *Rsc8 Scramble_1* | Positive | -28 | 0.017 | 5.1 | 0.22 | Yes |
| *Rsc8 Scramble_2* | Positive | -29.7 | 0.017 | 5.1 | 0.22 | Yes |
| *Rsc8 Scramble_3* | Positive | -27.9 | 0.017 | 5.1 | 0.22 | Yes |
| *Rsc8 Scramble_4* | Positive | -29 | 0.017 | 5.1 | 0.22 | Yes |
| *Mfg1 Scramble* | Negative | -21.9 | -0.001 | 0 | -0.3 | No |
| *Pub1 Scramble* | Negative | -43.5 | 0.058 | 0 | -0.27 | No |
| *Sro9 Scramble* | Negative | -63 | 0.106 | 0 | -0.14 | No |

**Supplementary Table S2:** Performances of the composition-based and SGnn approaches in the prediction of heat-induced recruitment of synthetic PrLDs to SG.

|  | Composition-based | SGnn |
| --- | --- | --- |
| **Specificity** | 1 | 0.95 |
| **Sensitivity** | 0.67 | 1 |
| **Accuracy** | 0.87 | 0.97 |
| **Precision** | 0.83 | 1 |
| **F1 Score** | 0.90 | 0.97 |
| **Matthews correlation coefficient** | 0.74 | 0.94 |
